# Lessons from Studies to Evaluate an Online 24-Hour Recall for Use with Children and Adults in Canada

**DOI:** 10.3390/nu9020100

**Published:** 2017-01-31

**Authors:** Sharon I. Kirkpatrick, Anne M. Gilsing, Erin Hobin, Nathan M. Solbak, Angela Wallace, Jess Haines, Alexandra J. Mayhew, Sarah K. Orr, Parminder Raina, Paula J. Robson, Jocelyn E. Sacco, Heather K. Whelan

**Affiliations:** 1 School of Public Health and Health Systems, University of Waterloo, Waterloo, ON N2L 3G1, Canada; 2 Department of Health Research Methods, Evidence, and Impact, McMaster University, Hamilton, ON L8S 4K1, Canada; gilsing@mcmaster.ca (A.M.G.); mayhewaj@mcmaster.ca (A.M.); praina@mcmaster.ca (P.R.); 3 McMaster Institute for Research on Aging, Hamilton, ON L8S 4L8, Canada; 4 Labarge Centre for Mobility in Aging, Hamilton, ON L8S 4K1, Canada; 5 Health Promotion, Chronic Disease and Injury Prevention, Public Health Ontario, Toronto, ON M5G 1V2, Canada; Erin.Hobin@oahpp.ca (E.H.); Sarah.Orr@oahpp.ca (S.K.O.); 6 Cancer Measurement, Outcomes, Research and Evaluation, Cancer Control Alberta, Alberta Health Services, Calgary, AB T2T 5C7, Canada; Nathan.Solbak@ahs.ca (N.M.S.); Paula.Robson@ahs.ca (P.J.R.); 7 Department of Family Relations and Applied Nutrition, University of Guelph, Guelph, ON N1G 2W1, Canada; angelaw@uoguelph.ca (A.W.); jhaines@uoguelph.ca (J.H.); 8 Department of Agricultural, Food and Nutritional Science, Faculty of Agricultural, Life and Environmental Sciences, University of Alberta, Edmonton, AB T6G 2R3, Canada; 9 Prevention and Cancer Control, Cancer Care Ontario, Toronto, ON M5G 2L7, Canada; jocelyn.sacco@cancercare.on.ca; 10 Health and Physical Education, Mount Royal University, Calgary, AB T3E 6K6, Canada; hwhelan@mtroyal.ca

**Keywords:** dietary intake, technology-enabled dietary assessment, 24-h recalls, Automated Self-Administered 24-h Dietary Assessment Tool, feasibility, validity, web-based

## Abstract

With technological innovation, comprehensive dietary intake data can be collected in a wide range of studies and settings. The Automated Self-Administered 24-h (ASA24) Dietary Assessment Tool is a web-based system that guides respondents through 24-h recalls. The purpose of this paper is to describe lessons learned from five studies that assessed the feasibility and validity of ASA24 for capturing recall data among several population subgroups in Canada. These studies were conducted within a childcare setting (preschool children with reporting by parents), in public schools (children in grades 5–8; aged 10–13 years), and with community-based samples drawn from existing cohorts of adults and older adults. Themes emerged across studies regarding receptivity to completing ASA24, user experiences with the interface, and practical considerations for different populations. Overall, we found high acceptance of ASA24 among these diverse samples. However, the ASA24 interface was not intuitive for some participants, particularly young children and older adults. As well, technological challenges were encountered. These observations underscore the importance of piloting protocols using online tools, as well as consideration of the potential need for tailored resources to support study participants. Lessons gleaned can inform the effective use of technology-enabled dietary assessment tools in research.

## 1. Introduction

Technological innovations in dietary assessment have been pursued to reduce researcher and respondent burden, while enabling the collection of high-quality dietary data [1,2,3,4,5]. One such innovation is the Automated Self-Administered 24-h (ASA24) Dietary Assessment Tool, developed by the U.S. National Cancer Institute [6]. In addition to allowing self-administration of 24-h recalls (24HRs), ASA24 incorporates automated coding and thus negates the need for coders. As a result, it is possible to collect 24HRs in studies in which this would previously have been cost-prohibitive. Studies conducted among adults in the US have suggested high rates of accuracy of recalled intake relative to true consumption, as well as comparability with interviewer-administered 24HRs [7]. Research also indicates high levels of acceptance of ASA24 among adults with Internet access [8]. Evaluations with children have highlighted challenges among younger children and pointed to the need for additional research to determine the age at which it is possible to use a self-administered tool such as ASA24 [9,10]. There has been little evaluation to date with older adults. 

A Canadian adaptation of ASA24 has been developed to reflect the Canadian food supply [11]. Modifications included adding foods and beverages unique to Canada and removing those not available in this country, changes to reflect differences in brand names in Canada compared to the U.S., and the addition of metric units. ASA24-Canada has been freely available since the spring of 2014 [11]. An updated interface, released in the fall of 2016, expanded usage to mobile devices in addition to laptop and desktop computers, provided the capacity to collect food records as well as recalls, and introduced a French language option [11]. As a result, ASA24 represents a potentially viable tool for the collection of dietary data in a range of research studies within the Canadian context.

Several studies to evaluate the feasibility and validity of ASA24 have been undertaken with Canadian populations, including preschoolers (with parental reporting), school-aged children, adults, and older adults. The objective of this paper is to discuss experiences in implementing ASA24 and lessons learned from these studies, with the aim of informing the use of technology-enabled dietary assessment tools with diverse samples. 

## 2. Materials and Methods 

We draw upon five studies to glean insights into the use of ASA24 in Canadian populations. One study conducted in a childcare setting assessed the capacity of parents to accurately recall preschool children’s intake. Two studies, one assessing feasibility and one assessing criterion validity, were conducted within schools with children in grades 5 through 8 (ages 10–13 years). Two studies, both with adults, examined the feasibility of the tool for use in samples enrolled in large cohort studies. Detailed methods and the main results from each of these studies in terms of validity and/or feasibility will be published elsewhere. In this paper, we provide a brief overview of the samples, recruitment, and protocols for administration of ASA24. Each study team provided an overview of their experiences using ASA24, which were synthesized according to key lessons learned. We highlight considerations based on specific study designs, contexts, and populations. 

### 2.1. Automated Self-Administered 24-Hour (ASA24) Dietary Assessment Tool 

ASA24 is a dynamic online interface that guides respondents through the completion of 24HRs using multiple passes and tailored portion size images [6]. The passes are adapted from the Automated Multiple-Pass Method [12]. Briefly, ASA24 prompts respondents to report eating occasions and search or browse categories for the foods and beverages consumed. Respondents are then queried about details such as preparation methods and portion sizes, as well as frequently forgotten foods and beverages. Researchers can choose to administer optional modules to collect details such as the location of eating occasions and the source of the main ingredients for the foods and beverages reported [6].

At the time of implementation of the studies, ASA24-Canada-2014, which relied upon the use of the Silverlight plug-in, was available. This plug-in, which is used by some Websites to display multimedia content and must be enabled on the computer on which ASA24 is being completed, is no longer supported by many commonly-used Internet browsers [13], which creates challenges for respondents depending on the browsers used. To avoid this issue, ASA24-2016 (which does not use the Silverlight plug-in) was used in multiple studies described here (Table 1). This allowed researchers to evaluate the interface reflecting the version of ASA24 available in Canada in the foreseeable future (the Canadian version of ASA24-2016 was released in October 2016). All studies were conducted in English. 

### 2.2. Overview of Feasibility and Validity Studies

Table 1 provides an overview of the five studies, with additional salient details provided below. 

#### 2.2.1. Observational Feeding Study among Toddlers with Parent Reporters (Study 1; S1)

To assess the criterion validity of ASA24 for capturing the intake of preschoolers (aged 2–5 years) using parental reporting, an observational feeding study (*n* = 40 parent-child dyads) was conducted. Methods were adapted from earlier U.S. research with adults [7] and involved data collection over 2-day cycles at a childcare centre. On day 1, researchers documented children’s lunch and snack intake. Parents (84% mothers and 16% fathers), and, in some cases other family members (e.g., siblings), joined the child participants for dinner at the centre. On day 2, parents returned to complete ASA24-Canada-2014 for the prior 24 h (including all meals, whether or not they were present) and a brief demographic questionnaire. This study received ethics approval from the University of Guelph Research Ethics Board (ref. 15JN028; approved 23 July 2015).

#### 2.2.2. School-Based Feasibility Study with Children (Study 2; S2)

A sigurechool-based study was conducted to examine ASA24 completion rates and user experiences among school-aged children (aged 10–13 years). A total of 294 students were recruited from 24 classrooms in eight schools in Ontario. Students with parental consent and who assented to participate were asked to complete ASA24-2016 for the past day, and a brief demographic and health survey. During data collection, challenges encountered by students completing ASA24-2016 were recorded by researchers. Students who provided valid email addresses (79% of the sample) were subsequently invited to complete ASA24 at home. After the at-home recall was completed or the window for completion had passed, an email invitation to complete a brief online survey about experiences using ASA24 was sent; items on this survey assessed specific features of students’ experiences with the ASA24 as well as their overall experience by asking: “Overall, how easy was it for you to complete the online food and nutrition recall?”. Response options included very difficult, difficult, neutral, easy, very easy, don’t know/unsure, and prefer not to answer. This study received ethics approval from Public Health Ontario’s Ethics Review Board (file 2015-073.01; approved 27 January 2016) and the University of Waterloo Office of Research Ethics (ref. 21155; approved 26 January 2016).

#### 2.2.3. School-Based Observational Feeding Study with Children (Study 3; S3)

**Figure 1:**
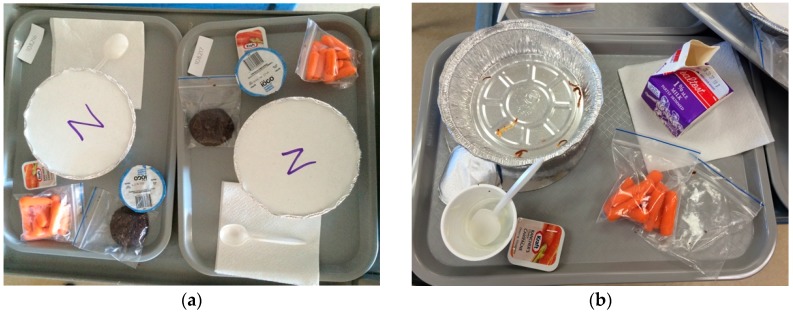
(**a**) Prepared trays served to children in school-based observational feeding study; (**b**) Plate waste collected for weighing after lunch in school-based observational feeding study.

A feeding study conducted in tandem with Study 2 was utilized to assess the criterion validity of ASA24 data collected from school-aged children (aged 10–13 years). Methods were adapted from U.S. research with adults [7]. Participation involved consuming a study-provided lunch (Figure 1) and completing ASA24 the following day, along with a demographic and health survey. A total of 98 students from eight classes in three schools participated on both days. For seven of eight classes, we used ASA24-2016. ASA24-Kids-2014 was employed in one grade 5 classroom to explore whether younger children were better able to report their intake using this version. This study received ethics approval from Public Health Ontario’s Ethics Review Board (file 2015-073.01; approved 27 January 2016) and the University of Waterloo Office of Research Ethics (ref. 21155; approved 26 January 2016). 

#### 2.2.4. Community-Based Feasibility Study with Adults (Study 4; S4)

To assess the feasibility of ASA24 for use with adults in an existing cohort, a sub-study was conducted among a random sample of Alberta’s Tomorrow Project (ATP) [14] participants, aged 36–82 years. Completion rates for up to four ASA24 administrations over four months, followed by one administration of the Canadian Diet History Questionnaire [15], were examined. Of 550 participants invited, 331 (29% men) provided consent and were asked to complete a short health and socio-demographic questionnaire. The first group (*n* = 140) of participants was asked to complete the first recall using ASA24-Canada-2014, but due to challenges with the Silverlight plug-in, subsequent 24HRs were completed using ASA24-2016. Up to three reminder emails were sent following the initial request to complete ASA24. Participants were asked about their experiences with ASA24 using items adapted from the System Usability Scale [16]. This study was approved by the Health Research Ethics Board of Alberta (Cancer Committee) (ID: 14-203; approved 24 August 2015).

#### 2.2.5. Community-Based Study with Older Adults (Study 5; S5)

Feasibility of ASA24 administration was tested in a sample of individuals in the Canadian Longitudinal Study on Aging [17] (aged 48–88 years) (the main aim of this sub-study was to examine the relative validity and reliability of the Short Diet Questionnaire (SDQ) [18] using the ASA24 data). Participants with high-speed Internet access were recruited during in-person visits to the study center; a subset of younger participants (48–67 years) was recruited over the phone. Participation involved completing four recalls using ASA24-Canada-2014 over three months. For participants recruited in person, the first recall was completed in the presence of a researcher. Remaining recalls for these participants and all recalls for those recruited by phone were completed at home, with up to five attempts. If participants were not able to complete ASA24 independently (after receiving assistance by phone or email), they were given the option to complete ASA24 with an interviewer over the phone. Experiences with ASA24 were assessed using items from the System Usability Scale [16]. This study received approval from the Hamilton Integrated Research Ethics Board (project number 0750; approved 26 October 2015). 

## 3. Results

### 3.1. Receptivity to Completing ASA24

Overall, participants within the studies were quite receptive to completing ASA24, even when they encountered technological challenges (elaborated upon below). Within the childcare setting (S1), participating families demonstrated a commitment to the study, with a low attrition rate: only 2 of 40 parents did not fully complete ASA24-Canada (both started the recall but did not complete all of the passes). In the school-based studies (S2 and S3), participating schools, teachers and children were enthusiastic about the study (particularly the validity study for which the provision of a free lunch was an exciting incentive for students). Within the classroom, students were generally willing to engage with the ASA24 system and the vast majority of those with parental consent and who assented to participate at least started the recall. Of students participating in the feasibility study (S2) who submitted a user experience survey (14%), the majority indicated they found completing ASA24 “very easy”, “easy”, or “neutral”. However, a number of children in that study either did not provide valid email addresses (21%) or attempt to complete the at-home ASA24 (62%).

In the feasibility study of adults (S4), participants also appeared keen to complete ASA24. Several participants contacted the study centre to inform staff about upcoming periods of unavailability (e.g., due to travel), seeking to remain part of the study nonetheless. For recalls conducted using ASA24-Canada-2014, participants spent considerable amounts of time navigating issues with the Silverlight plug-in, highlighting their willingness to complete the study elements. Further, despite some frustrations about the time involved in completing 24HRs, participants made it clear they wanted to remain in the study. During the course of the 16-week study, 50 participants (15% of the sample) actively withdrew; just under half did not provide a reason, while approximately one-fifth cited challenges completing ASA24 (other reasons included being too busy, travel, and too much time required to complete the assessments). 

In the study of older adults (S5), respondents were eager to learn how to complete ASA24 and indicated they did not mind spending time doing so. Some older adults viewed this as a way to maintain and enhance their technological skills (though the extent to which study staff could assist in this regard was limited due to feasibility). Participants also shared accounts of spouses, children and friends helping them navigate technological issues to allow completion of ASA24. Across the age and sex strata (younger versus older, men versus women), fewer than 20% of those who completed the first recall did not complete the full study. In this study, recruiting participants in person appeared to have a beneficial effect on completion compared to recruiting by phone, perhaps due to the upfront time investment and rapport established during the first study visit and/or the training on the use of ASA24, which may have increased motivation and/or mitigated technological issues. 

### 3.2. User Experiences with the ASA24 Interface

Given the self-administered nature of ASA24, some participants experienced challenges in navigating the passes. In particular, participants were sometimes unclear as to the next step to complete. For example, some students in the school-based studies (S2 and S3) assumed they were finished after describing a single eating occasion or after reporting the foods and drinks consumed at multiple meals but without providing details about these foods and drinks. In these cases, researchers encouraged students to continue by prompting them to “keep clicking, try your best, scroll down” (depending on the screen size on the device used, the entire frame may not show on a single screen, requiring scrolling to reach the next step). Further, some students did not understand that the search function required entering a single term (e.g., “banana”) at a time, sometimes entering multiple items (e.g., “banana cereal orange juice”), which does not return the desired results. Some did not understand how to filter using ASA24’s option for sorting search results by category. As well, some students had difficulty interpreting response options to the question “How much did you actually eat?”, perceiving the portion size images to show the plate or bowl once they were finished eating (i.e., an almost empty plate was interpreted to mean they finished most of their meal rather than representing the amount they actually ate). Finally, the language used is not necessarily child-friendly (e.g., “pat” of butter), leading to some confusion. 

In the feasibility study of adults (S4), some respondents had difficulty finding the foods they wanted to add, particularly if they searched for a generic food item (e.g., “bread”) that sometimes resulted in a very long list of foods to consider. In the community-based study of older adults (S5), older participants were observed to be more likely to browse for foods using the categories (rather than searching), resulting in longer completion times. Some older participants had difficulties understanding how the search functioned and how to navigate the results returned, as well as how to move selected foods to their recall. Younger adults within this study (S5) appeared more comfortable using the search, speeding up the entry of foods. However, older adults were observed to be more patient than younger adults with the repetition in completing the multiple passes. 

In the study of preschoolers (S1), some parents recalled only the dinner meal for which they were present with their children, despite ASA24’s repeated prompts to report all foods and drinks consumed the prior 24-h period. This suggests that parents perhaps did not understand they were to report all eating occasions, even those for which they were not present, or that they may not have felt confident reporting meals for which they did not have detailed knowledge.

### 3.3. Technological Challenges and Provision of Support

For studies using ASA24-Canada-2014, challenges were encountered related to the Microsoft Silverlight plug-in. Additionally, some participants in the various studies experienced “freezing” of ASA24 midway through completion, sometimes resulting in the loss of data. It was unclear whether this arose due to problems with Internet connectivity or other issues. 

For the childcare centre- and school-based studies (S1, S2 and S3), researchers were available on site. Given the study objectives related to feasibility and validity, participants were encouraged to complete ASA24 independently to the extent possible in the face of issues that did not require intervention to address technological issues, such as problems entering passwords or “freezing”. In the community-based studies of adults and older adults, for which recalls were completed at home, there were relatively frequent requests for assistance from study staff. 

In the feasibility study of adults (S4), participants were not provided with prior training in the use of ASA24, but assistance was available via phone or email. Over half of the participants in this study contacted the study centre for help at least once, and over one in ten made three or more contacts for assistance. Password issues, specifically forgotten or misplaced login credentials, were common. Staff were generally able to assist with these issues, but in some situations, had difficulty verbally describing non-alphanumeric characters used in passwords (e.g., @, !, and particularly & (ampersand)). The capacity to assist with requests for help by email or phone depended upon participants’ computer literacy, as well as the nature of the technical problem. As the study progressed, staff became familiar with common issues and, in some cases, sought the advice of the information technology team to find solutions that could be communicated to participants by phone or email. Staff members reported anecdotally that informing participants that similar issues had been encountered by others seemed to provide comfort and increased willingness to continue with the study. 

In the community-based study of older adults (S5), most recalls were completed at home and assistance was available by email and telephone. Some participants experienced problems copying and pasting the link to ASA24 and with login credentials. It was observed that technological troubleshooting capacity was more limited in older participants compared to younger participants. However, compared to younger adults within the sample, older participants were willing to spend more time troubleshooting. Further, some older adults reported vision problems and used special computers and software that did not always display ASA24 correctly. Participants who were unable to complete recalls independently were given the opportunity to do so over the phone. Using ASA24 as an interviewer guide allowed research staff to collect recall data in a standardized manner. Most telephone-administered recalls required less than 20 min to complete; this was deemed a reasonable investment in terms of study resources to avoid drop out. Many older participants reported having smart phones or tablets, which they were more comfortable using than laptop/desktop computers. This study used ASA24-2014 but this observation speaks to the potential for ASA24-Canada-2016, which can be completed on mobile devices, to expand uses of ASA24.

### 3.4. Time to Complete

In the study of preschoolers (S1), time to complete ASA24 typically fell between 25 and 45 min (median, 35 min). Given that data collection was conducted during drop-offs to the childcare centre, some parents found it difficult to devote the time to complete ASA24-Canada due to work schedules. Some exceptions were made to allow parents to complete the recall later in the day from another location (e.g., work, home); this led to some technological challenges with the Silverlight plug-in. Time to complete ASA24 also emerged as a challenge within the school setting (S2 and S3), in which limited classroom time (a 50-min period, with ~35–40 min for ASA24) was allotted to the study activities. For school-aged children, the median time for completion was 35 min for the feasibility study and 34 min for the validity study (few students in the validity study completed ASA24-Kids, thus, these estimates are limited to ASA24-2016). Younger students were observed to take longer and many did not finish the recalls in the given time. Given our research interests, we opted to enable the ASA24 source module, which asks about the source (e.g., grocery store) of the main ingredients for each food and beverage; this likely added significantly to completion times. 

In the feasibility study of adults (S4), the median time to complete recalls was 41 min; this decreased with successive recalls. The time to complete in the sample of older adults (S5) ranged from 15 min to 2 h; we do not report median completion times for this study since both self-administered and interviewer-administered ASA24 recalls were completed (these results will be detailed elsewhere). Based on interviewer-administered ASA24 recalls completed over the phone, time to complete appeared to depend on how complex the participant’s meals were, how long it took the participant to remember details, and the extent to which he or she discussed the details of what was consumed. For initial recalls completed in the presence of a researcher, it was observed that the reaction and processing time of older adults for each step was considerably longer than for younger adult participants. 

### 3.5. Costs Associated with the Use of ASA24 in Research

The ASA24 tool itself is freely accessible to researchers to use. However, the implementation of ASA24 does require resources. For example, in the studies described here, there were costs associated with supporting participants (e.g., provision of assistance by telephone and email, completion of initial recalls in the presence of a researcher, interviewer-administration for some participants). As with any study, there are also resources associated with recruitment, contacting and monitoring participants, and data analyses. The costs associated with collecting recalls using ASA24 will vary in relation to sample size, the number of recalls to be completed, the level and types of support provided, and other study-specific factors. 

### 3.6. Considerations in Conducting ASA24 Studies in Institutional Settings

The study of parents’ ability to report their children’s intakes was conducted within a childcare setting (S1) and provided the opportunity to assess the accuracy of parental reporting for meals both for which the parent was and was not present. However, as noted above, time challenges were sometimes an issue for busy parents. For the studies of school-aged children (S2 and S3), working within the school setting meant numerous constraints, including a lack of control over scheduling and time allotment for ASA24 completion, as well as interruptions (e.g., due to announcements). Further, some classroom computers had Internet Explorer only, which seemed to perform less optimally than Chrome for ASA24. Lastly, using email as a means of contacting students recruited within the school setting was not effective in terms of completion of subsequent at-home recalls. 

### 3.7. Considerations in Administering ASA24 in Community-Based Samples

Practical issues influencing participants’ experiences of ASA24 emerged in the community-based studies. For example, the scheduling of recalls had a bearing on the availability of support for participants. In the feasibility study of adults (S4), support available by phone or by email was limited to office hours on weekdays. Thus, participants who required assistance on a Friday evening did not receive support until Monday morning, by which time the window for ASA24 completion had closed. Researchers can choose to allow unscheduled recalls (the participant can log in at any time to complete a recall) or use the scheduled recall setting (the participant must log in on a specific day to complete a recall). With the scheduled setting, some participants reported they would have liked to complete the recall, but owing to time constraints or technological issues for which they needed assistance, they missed the window. Changing to an unscheduled setting increased the proportion of participants who completed recalls, but increased the risk of reactivity as participants knew they were to complete a recall but may not have done so until the following day, providing an opportunity to modify their intake for the recalled period. Each of the studies involving at-home completion of ASA24 included multiple attempts as a means of increasing completion rates given that sometimes participants were willing to complete recalls but unable to do so in a given time period. 

Misplaced passwords also posed challenges. In the feasibility study with adults (S4), passwords were mailed to participants in a letter shortly after they consented to participate, and in some cases, may have arrived up to three weeks prior to the first invitation to complete a recall. Participants who lost passwords had to contact study staff before they could proceed with ASA24. Passwords were also an issue for some participants in the study with older adults (S5).

### 3.8. Unique Issues in Validity Feeding Studies

**Figure 2:**
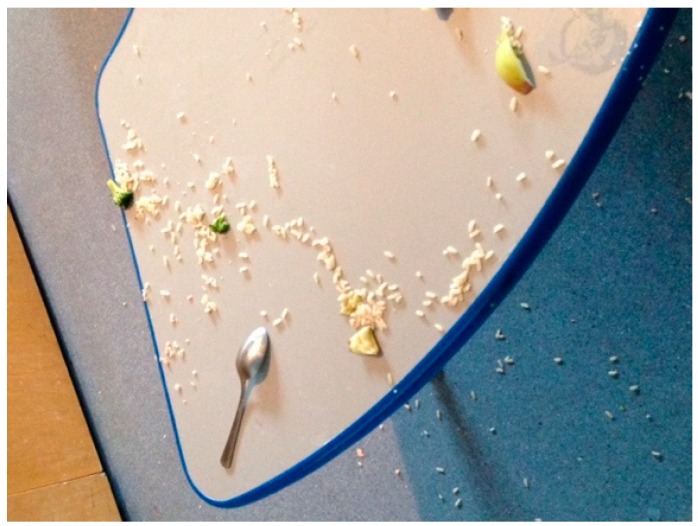
Spillage of rice and other items served to a preschooler in Study 1 (observational feeding study with preschoolers and parent reporters).

**Figure 3:**
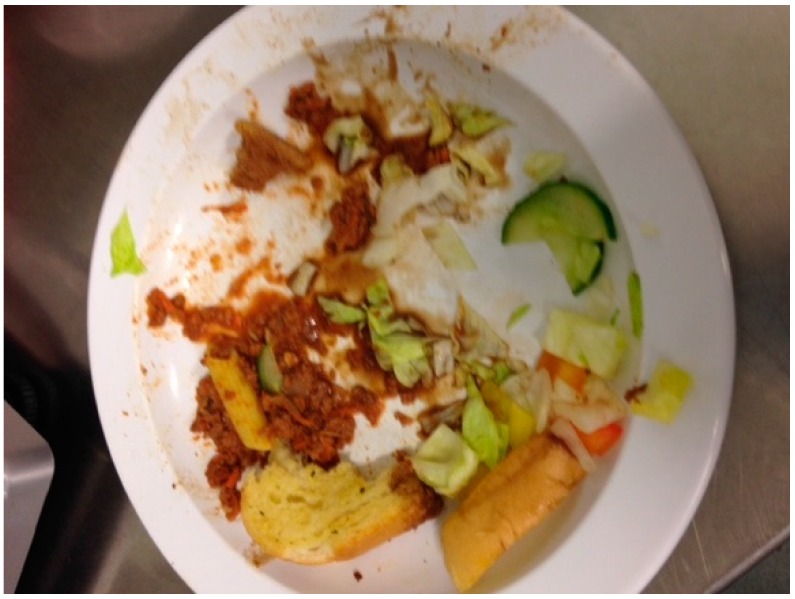
Example of plate waste in Study 1, with various foods mixed together (observational feeding study with preschoolers and parent reporters).

Both feeding studies were conducted with children, one with preschoolers (with parent reporters) and the second with school-aged children. The childcare setting (S1) allowed for control over study food: researchers were present during meal preparation and ensured items were prepared according to the study protocol and accurately weighed prior to serving. Collecting and weighing plate waste was challenging given that young children can be messy eaters. For example, the research team had to collect spillage from the floor and bibs (Figure 2). In addition, young children tend to mix foods together, making it difficult in some cases to decipher and weigh plate waste (Figure 3). 

In the feeding study with school-aged children (S3), conducting the study within classrooms offered the advantage of completing data collection with a number of children simultaneously, but this also posed challenges. On day one, despite being asked to leave all remaining items including packaging on trays and the watchful eyes of the researchers, some students took items with them for the recess break that followed lunch. Further, because participating schools were geographically dispersed, foods and drinks were ordered from a central caterer but delivered by local operators. Variation in items (e.g., standardization of carrots by number rather than weight, different brands of yogurt) complicated the study protocol in terms of calculating amounts consumed. Spillage and mixing of foods were not major problems as in the study of preschoolers and researchers monitored the lunch meal to avoid, or at least minimize, trading of foods and drinks.

## 4. Discussion

**Figure 4:**
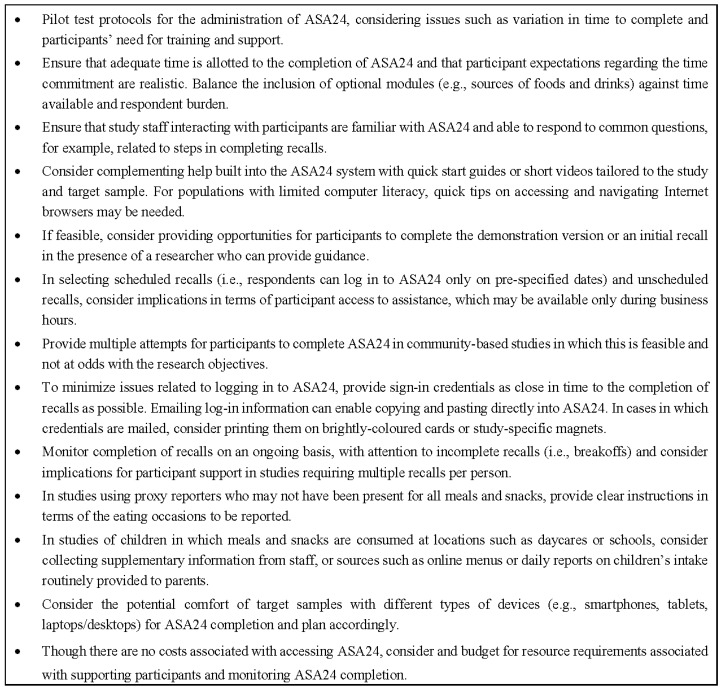
Considerations in implementing ASA24 in research studies.

Comparing and contrasting the experiences of researchers evaluating ASA24 in diverse samples provides insights into challenges that might be commonly encountered in research using technology-based tools and potential strategies to mitigate these. Overall, we found participants were receptive to completing ASA24, and in some cases, willing to invest significant effort to do so. However, technology does not negate all challenges in collecting dietary intake data and indeed, may introduce new issues, for example, due to the need for computer literacy. As a result, it is critical to carefully consider the implementation of technology-based approaches into research and the potential need for tailored supports for study participants; Figure 4 summarizes considerations emerging from the experiences of the research teams leading these five studies in implementing ASA24.

The research teams observed that the ASA24 interface and the steps in completing recalls were not necessarily intuitive for all respondents. Generally, challenges appeared greater for younger versus older children, presumably due to less developed cognitive skills. Further analysis of the validity study data for school-aged children will provide insights into the age at which children might be expected to complete ASA24 independently. As well, older versus younger adults sometimes exhibited less comfort with computers and the Internet. In the study of older adults, having participants complete an initial recall in the presence of a staff member appeared to be beneficial in supporting future independent completion of ASA24. 

These studies also illustrated that the time to complete ASA24 can be variable. This was evident in those that included both younger and older children and younger and older adults, respectively, with younger children and older adults needing more time to complete ASA24 than others. Pilot testing is critical to ensure that the time allotted to ASA24 within a study is sufficient. Attention to the optional modules enabled within ASA24 is important from the perspective of time. In the school-based studies, the source module was enabled to allow collection of information on acquisition of foods from fast food restaurants, for example. However, given that this module results in an extra probe for most foods and drinks reported, it is likely to have added quite significantly to completion times. As with any assessment tool, it is necessary to consider the extent of data to be collected along with the respondent burden and other study constraints.

In some cases, technological challenges encountered in these studies relate to factors no longer relevant to ASA24, including problems with the Silverlight plug-in used in the 2014 and earlier versions, now superseded by ASA24-2016. Instances of “freezing” may have been related to bugs in the 2016 version shortly after release that have since been remedied. However, with the use of web-based tools, it is virtually impossible to prevent all possible technology-related issues given that the reliability of Internet connections can vary and completion of ASA24 can be affected by issues outside the system itself. This is likely a particular issue in studies including participants who may not have access to high-speed Internet. Given that it is difficult to predict all potential issues that participants may encounter when interacting with technology, the provision of some level of support from research staff is likely to be important in most studies. Comfort with different devices is also a consideration; for example, some older participants reported having smart phones or tablets, which they were more comfortable using than laptops or desktop computers. ASA24-2016 is operational on both mobile devices and laptops and desktops, offering flexibility to researchers and study participants.

## 5. Conclusions

Using ASA24, researchers can collect dietary intake data in a broad range of studies, with almost instant access to coded nutrient and food group output. Biomarker-based studies have shown that 24HRs capture dietary intake data with less bias than do food frequency questionnaires [19,20]. The availability of ASA24-Canada supports a shift toward 24HR methodology by overcoming challenges associated with interviewer-administered recalls. These include recruiting and funding trained personnel to conduct interviews, clean data, and assign food codes to allow linkage with nutrient and food group databases. In the studies described here, ASA24 appeared to be acceptable and feasible overall. However, challenges associated with self-administration and technology were encountered, highlighting the need for piloting and attention to supports to enable completion of web-based tools in samples with variable cognitive skills and computer literacy. 

## Figures and Tables

**Table 1:** Overview of methods used in five studies examining validity or feasibility of ASA24.

Study Design	Objectives	Sample	ASA24 Version	Protocol for ASA24 Administration	Supports for ASA24 Completion
S1: Observational feeding study among toddlers with parent reporting	To assess the criterion validity of ASA24 for capturing preschooler’s intake using parent reporting	40 dyads of preschoolers (aged 2–5 years) and their parents were recruited from the Child Care and Learning Centre, University of Guelph, Ontario	ASA24-Canada-2014	Parents completed ASA24 on the day following monitoring of children’s lunch, snack, and dinner intake	Independent completion by parents; researchers present to address technological issues
S2: Feasibility study with school-aged children	To assess ASA24 completion rates at school and at home, as well as user experiences and barriers to completion, among children in grades 6–7	294 students (aged 10–13 years) were recruited from 8 schools in Ontario	ASA24-2016 (US version)	Children completed one ASA24 recall at school during class time and were invited by email to complete a second recall at home	Independent completion by children; researchers present to address technological issues
S3: Observational feeding study with school-aged children	To assess criterion validity of ASA24 for capturing intake among children in grades 5–8	98 students (aged 10–13 years) were recruited from 3 schools in Ontario	ASA24-2016 (US version); ASA24-2014-Kids used with one grade 5 class	Children completed ASA24 at school during class time on the day following monitoring of lunch intake	Independent completion by children; researchers present to address technological issues
S4: Feasibility study with adults	To assess ASA24 completion rates and variables associated with completion among adults	331 existing members (aged 36–82 years) of Alberta’s Tomorrow Project were recruited	ASA24-Canada-2014 and ASA24-2016 (US version)	Adults were prompted by email to complete ASA24 four times over a 4-month period	Independent completion by participants; assistance available by phone or email
S5: Community-based study with older adults	To examine feasibility of ASA24 for use with older adults and assess the validity and reliability of a short diet questionnaire compared to ASA24	264 existing members (aged 48–88 years) of the Canadian Longitudinal Study on Aging were targeted for recruitment	ASA24-Canada-2014	Adults were invited to complete ASA24 four times over a 3-month period; recalls completed at a study centre, independently at home, or over the phone	Initial recalls for most participants completed in presence of a researcher; assistance for at home recalls available by email and phone

ASA24, Automated Self-Administered 24-h Dietary Assessment Tool.

## References

[1] Rollo M.E., Williams R.L., Burrows T., Kirkpatrick S.I., Bucher T., Collins C.E. (2016). What are they really eating? A review on new approaches to dietary intake assessment and validation. Curr. Nutr. Rep..

[2] Kirkpatrick S.I., Collins C.E. (2016). Assessment of nutrient intakes: Introduction to the Special Issue. Nutrients.

[3] Thompson F.E., Subar A.F., Loria C.M., Reedy J.L., Baranowski T. (2010). Need for technological innovation in dietary assessment. J. Am. Diet. Assoc..

[4] Shriver B.J., Roman-Shriver C.R., Long J.D. (2010). Technology-based methods of dietary assessment: Recent developments and considerations for clinical practice. Curr. Opin. Clin. Nutr. Metab. Care.

[5] Thompson F.E., Subar A.F., Coulston A.M., Boushey C.J., Ferruzzi M.G. (2013). Dietary Assessment Methodology. Nutrition in the Prevention and Treatment of Disease.

[6] Subar A.F., Kirkpatrick S.I., Mittl B., Zimmerman T.P., Thompson F.E., Bingley C., Willis G., Islam N.G., Baranowski T., McNutt S. (2012). The Automated Self-Administered 24-h dietary recall (ASA24): A resource for researchers, clinicians, and educators from the National Cancer Institute. J. Acad. Nutr. Diet..

[7] Kirkpatrick S.I., Subar A.F., Douglass D., Zimmerman T.P., Thompson F.E., Kahle L.L., George S.M., Dodd K.W., Potischman N. (2014). Performance of the Automated Self-Administered 24-h Recall relative to a measure of true intakes and to an interviewer-administered 24-h recall. Am. J. Clin. Nutr..

[8] Thompson F.E., Dixit-Joshi S., Potischman N., Dodd K.W., Kirkpatrick S.I., Kushi L.H., Alexander G.L., Coleman L.A., Zimmerman T.P., Sundaram M.E. (2015). Comparison of interviewer-administered and automated self-administered 24-h dietary recalls in 3 diverse integrated health systems. Am. J. Epidemiol..

[9] Baranowski T., Islam N., Baranowski J., Martin S., Beltran A., Dadabhoy H., Adame S., Watson K.B., Thompson D., Cullen K.W. (2012). Comparison of a Web-based versus traditional diet recall among children. J. Acad. Nutr. Diet..

[10] Diep C.S., Hingle M., Chen T.-A., Dadabhoy H.R., Beltran A., Baranowski J., Subar A.F., Baranowski T. (2015). The Automated Self-Administered 24-h Dietary Recall for children, 2012 version, for youth aged 9 to 11 years: A validation study. J. Acad. Nutr. Diet..

[11] ASA24-Canada. http://asa24.ca/index.html.

[12] Blanton C.A., Moshfegh A.J., Baer D.J., Kretsch M.J. (2006). The USDA Automated Multiple-Pass Method accurately estimates group total energy and nutrient intake. J. Nutr..

[13] ASA24 Known Issues & Workarounds. https://epi.grants.cancer.gov/asa24/resources/issues.html.

[14] Robson P.J., Solbak N.M., Haig T.R., Whelan H.K., Vena J.E., Akawung A.K., Rosner W.K., Brenner D.R., Friedenreich C.M., Cook L.S. (2016). Design, methods and demographics from phase I of Alberta’s Tomorrow Project cohort: A prospective cohort profile. CMAJ Open.

[15] Csizmadi I., Boucher B.A., Lo Siou G., Massarelli I., Rondeau I., Garriguet D., Koushik A., Elenko J., Subar A.F. (2016). Using national dietary intake data to evaluate and adapt the US Diet History Questionnaire: The stepwise tailoring of an FFQ for Canadian use. Public Health Nutr..

[16] Bangor A., Kortum P., Miller J.A. (2008). The system usability scale (SUS): An empirical evaluation. Int. J. Hum.-Comput. Interact..

[17] Raina P.S., Wolfson C., Kirkland S.A., Griffith L.E., Oremus M., Patterson C., Tuokko H., Penning M., Balion C.M., Hogan D. (2009). The canadian longitudinal study on aging (CLSA). Can. J. Aging.

[18] Shatenstein B., Payette H. (2015). Evaluation of the relative validity of the Short Diet Questionnaire for assessing usual consumption frequencies of selected nutrients and foods. Nutrients.

[19] Freedman L.S., Commins J.M., Moler J.E., Willett W., Tinker L.F., Subar A.F., Spiegelman D., Rhodes D., Potischman N., Neuhouser M.L. (2015). Pooled results from 5 validation studies of dietary self-report instruments using recovery biomarkers for potassium and sodium intake. Am. J. Epidemiol..

[20] Freedman L.S., Commins J.M., Moler J.E., Arab L., Baer D.J., Kipnis V., Midthune D., Moshfegh A.J., Neuhouser M.L., Prentice R.L. (2014). Pooled results from 5 validation studies of dietary self-report instruments using recovery biomarkers for energy and protein intake. Am. J. Epidemiol..

